# Alien plant invasions and native plant extinctions: a six-threshold framework

**DOI:** 10.1093/aobpla/plw047

**Published:** 2016-08-02

**Authors:** Paul O. Downey, David M. Richardson

**Affiliations:** ^1^Institute for Applied Ecology, University of Canberra, ACT 2601, Australia; ^2^Centre for Invasion Biology, Department of Botany and Zoology, Stellenbosch University, Private Bag X1, Matieland 7602, South Africa

**Keywords:** Biological invasions, conservation, declining populations, extinction trajectory, invasive plant species, threshold breaches

## Abstract

Biological invasions are widely acknowledged as a major threat to global biodiversity. Species from all major taxonomic groups have become invasive. The range of impacts of invasive taxa and the overall magnitude of the threat is increasing. Plants comprise the biggest and best-studied group of invasive species. There is a growing debate; however, regarding the nature of the alien plant threat—in particular whether the outcome is likely to be the widespread extinction of native plant species. The debate has raised questions on whether the threat posed by invasive plants to native plants has been overstated. We provide a conceptual framework to guide discussion on this topic, in which the threat posed by invasive plants is considered in the context of a progression from no impact through to extinction. We define six thresholds along the ‘extinction trajectory’, global extinction being the final threshold. Although there are no documented examples of either ‘in the wild’ (Threshold 5) or global extinctions (Threshold 6) of native plants that are attributable solely to plant invasions, there is evidence that native plants have crossed or breached other thresholds along the extinction trajectory due to the impacts associated with plant invasions. Several factors may be masking where native species are on the trajectory; these include a lack of appropriate data to accurately map the position of species on the trajectory, the timeframe required to definitively state that extinctions have occurred and management interventions. Such interventions, focussing mainly on Thresholds 1–3 (a declining population through to the local extinction of a population), are likely to alter the extinction trajectory of some species. The critical issue for conservation managers is the trend, because interventions must be implemented before extinctions occur. Thus the lack of evidence for extinctions attributable to plant invasions does not mean we should disregard the broader threat.

## Abstract (precis)

Currently there is debate around whether the outcome of alien plant invasions is the extinction of native plants. We refocus the debate around a series of thresholds along an extinction trajectory where extinction is the end point. We identify and discuss a range of factors that influence our ability to state that extinction has occurred and the role of these factors in determining breaches of the other thresholds along the trajectory. Over-emphasis on the end point of the extinction trajectory ignores strong evidence for declines and breaches of other thresholds along the trajectory which are critical for understanding and managing the threat.

## Introduction

Plants contribute substantially to the global problem of biological invasions, both in terms of the number of species (see Pyšek *et al.* 2008) and their influences on ecosystems, especially on fire regimes (Brooks *et al.* 2004; Gaertner *et al.* 2014), nutrient cycling (Ehrenfeld 2010; also see below), ecosystem services (e.g. van Wilgen *et al.* 2008) and geomorphology (Fei *et al.* 2014). They also alter successional rates and trajectories (e.g. Potgieter *et al.* 2014). Islands have been particularly severely affected (Pyšek *et al.* 2012). Whereas invasive species in some other taxonomic groups have clearly contributed substantially to global extinctions (e.g. vertebrate predators causing rapid extinctions of native animals; Dickman 1996), the link between alien plant invasions and extinction of native plant species is much less clear (e.g. Bellard *et al.* 2016). This is in part because of a lack of appropriate data (both in terms of the measures used and the timeframes required to state conclusively that extinctions have occurred), and the lack of unambiguous examples of extinctions caused by alien plants, and the counteracting effects of the management of invasive plants.

As a result, there has been much debate about the potential for alien plant invasions to cause extinctions of native plant species. This has raised questions on whether expensive management interventions to control invasive plants to safeguard biodiversity are justified. For example:

- Sax and Gaines (2008) write: ‘Why so few plant species have been lost is somewhat of a mystery, particularly considering the thousands of exotic plant species that have been introduced to islands’;
- Sagoff (2005) contends that ‘there is no evidence that non-native species, especially plants, are significant causes of extinction, except for predators in certain lakes and other small island-like environments’;
- Several studies of alien plants have failed to detect direct impacts on native species (e.g. Anderson 1995; Skurski *et al.* 2013; Davis *et al.* 2015; Thomas and Palmer 2015)—but see Hulme *et al.* (2015).
- Gurevitch and Padilla (2004) in their review ‘Are invasive species a major cause of extinctions?’ conclude that ‘the generalization that alien species are playing a widespread role in extinctions is, to date, too unspecific to be either accurate or useful’—but see Ricciardi (2004).

The full dimensions of the impacts of plant invasions are inherently difficult to determine experimentally. Consequently, most publications dealing with negative effects of plant invasions on native species use surrogates like space-for-time and time-sequence (Richardson *et al.* 1989; Thomaz *et al.* 2012; Rejmánek 2012), or the effects are inferred (Miller and Gorchov 2004). Many studies use measures that are unlikely to unequivocally demonstrate extinctions. For example, information from field studies typically shows that alien plant species increase species richness (e.g. Fridley *et al.* 2007; Thomas and Palmer 2015). The fact that non-natives add to the number of species in a given area is sometimes raised to counter the argument that plant invasions have a negative impact on biodiversity (Sax and Gaines 2008; Thomas and Palmer 2015); increased species introductions increase biodiversity (including potentially generating new taxa through hybridization; Thomas 2015) and, therefore, that they do not merit concern as a global threat to biodiversity (see Richardson and Ricciardi 2013). Although such studies raise many interesting ecological questions, for example whether plant communities are ever saturated (Stohlgren *et al.* 2008; Bennett *et al.* 2012), they do not address the broader problem that some species experience declines.

Plant introductions around the world have clearly stimulated hybridization in many plant taxa, probably resulting in higher hybridization rates in recent centuries as suggested by Thomas (2015). Such hybridisations have, however, been identified as a threat to many native species (e.g. Daehler and Strong 1997). Indeed, we suggest that increases in biodiversity due to hybridization are likely to be trivial compared to the attrition in biodiversity wrought by invasive plant species. Also, ‘snapshot’ counts of species richness clearly do not allow for a meaningful quantification of impact nor can they demonstrate extinction, especially when the potential for persistence of many species recorded over long time periods is not considered (Richardson and Ricciardi 2013).

The studies outlined above typically fail to consider evidence that could contribute to extinctions in the long-term (e.g. local losses and range contractions: Ricciardi 2004), or the importance of population losses to species survival (Ricciardi 2004), and/or ignore the many studies that demonstrate such extinction trajectories, which may take hundreds of years to culminate in extinction (see Gilbert and Levine 2013). The lack of evidence for extinctions should surely not be justification for inaction or a change in emphasis in management (e.g. Simberloff 2005), and the lack of scientific certainty should not prevent the implementation of measures to mitigate the problem (Blossey *et al.* 2001).

In our view, the debate around the effect of plant invasions on the status of native plant biodiversity has yet to be framed in a manner that is conducive to finding a helpful solution. This paper aims to refocus the debate and guide further discussion on this topic by first considering extinction as the end point of a series of events that occur along the extinction trajectory. We believe that deliberation on whether species are progressing along the extinction trajectory is more helpful for assessing impacts and for guiding the management of alien plants than focusing on whether the end of the extinction trajectory has been reached. We identify five thresholds in addition to extinction which we believe should be used to reframe the debate on the impact of alien plants on native plants to produce more helpful guidance for conservation.

## Definition of extinction

The framework developed in this paper is underpinned by the standard definition of extinction used by the International Union for Conservation of Nature (IUCN) in their ‘Red list of threatened species’. An *extinct species* is here defined as one for which no individuals have been recorded, despite exhaustive surveys over a period that is appropriate for the life cycle of the species in habitats where the species could be expected to occur within its native range (IUCN 2014). For many years, the IUCN used a 50-year rule for the timeframe, but this rule has been tailored to the target species. Nonetheless, there is considerable discussion in conservation agencies about how one should determine that the last individual has died and the timeframe over which searching is required. Such discussions are based on the degree of uncertainty between an apparent ‘absence’ of the species and a lack of survey effort combined with the data needed to determine all individuals have been lost (see Boakes *et al.* 2016). Seed dormancy and seed banks in plants complicate the task of assessing whether every individual has been lost (see below).

## Conceptual framework: six thresholds on the extinction trajectory

**Figure 1.**
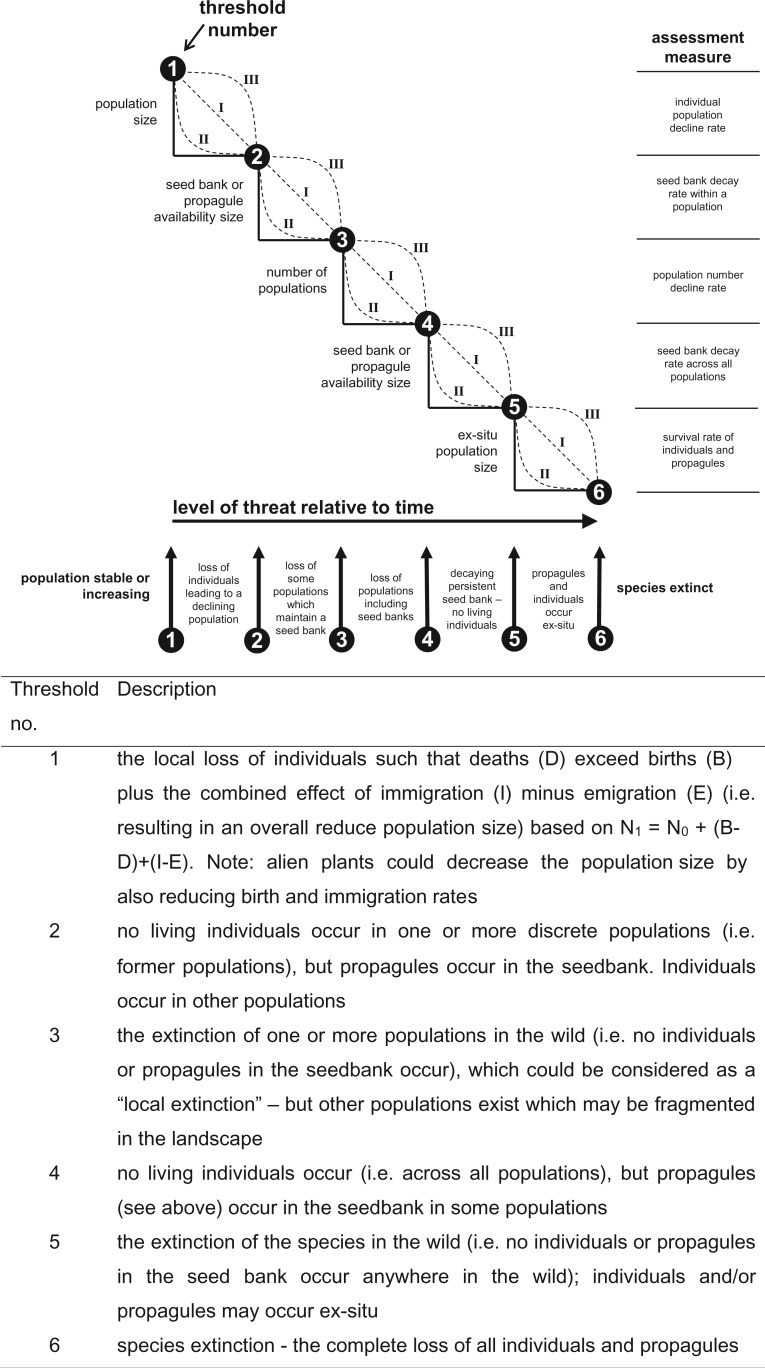
The six extinction trajectory thresholds. Three models (I, II and III) illustrate the transition a species may take between each threshold. Examples of the assessment measures required to demonstrate that threshold breaches have occur are shown.

The extinction of a species is the end point of a declining population or extinction trajectory. Although extinction is a critical point on the trajectory, other key points along the trajectory are also crucial. The extinction trajectory of a plant species encompasses six key points or thresholds (see Fig. 1), these being: ***Threshold* 1**—the local loss of individuals (deaths) at a rate that exceeds births plus the combined effect of immigration minus emigration (i.e. resulting in an overall declining population); ***Threshold* 2**—no living individuals occur in one or more discrete populations (i.e. former populations)—this may also include genotypes, but propagules occur in the seed bank (including below-ground dormant vegetative parts of a plant (e.g. *Epipactis albensis* may survive without any above-ground organs for 11 years (Rydlo 1995) and *Scirpus maritimus* for up to 25 years (Squires and van der Valk 1992)). Individuals occur in other populations; ***Threshold* 3**—the extinction of one or more populations in the wild (i.e. no individuals or propagules in the seed bank (see above) occur), which could be considered as a local extinction—but other populations exist which may be fragmented in the landscape; ***Threshold* 4**—no living individuals occur (i.e. across all populations), but propagules (see above) occur in the seed bank in some populations; ***Threshold* 5**—the extinction of the species in the wild (i.e. no individuals or propagules in the seed bank occur anywhere in the wild); individuals and/or propagules may occur ex-situ (i.e. germplasm, seeds in storage or individuals in cultivation); and ***Threshold 6***—species extinction—the complete loss of all individuals and propagules.

We use these six thresholds as the foundation for a conceptual framework to illustrate that although there is no evidence of native plant extinctions (i.e. at Threshold 6) that are entirely or directly attributable to alien plant invasions, there is abundant evidence that plant invasions are driving native plants across other thresholds. Further consideration of these six thresholds reveals that alien plants are unlikely to pose an on-going threat between Thresholds 5 and 6 (i.e. between extinct in the wild and global extinction), except in instances where Threshold 5 does not occur (i.e. there are no individuals or propagules *ex situ*).

### Transition between thresholds

The manner and processes by which individual native plant species transition towards a specific threshold (i.e. progress along the extinction trajectory) can take many forms or shapes (see models I, II and III in Fig 1, although a linear response (model I) is unlikely to occur). Species might exhibit different shaped trajectories for different thresholds. For example, a plant species with highly dormant seeds that is highly sensitive to an alien plant invasion might exhibit a model II trajectory while transitioning between Thresholds 1 and 2 (rapid population decline), and then exhibit a model III trajectory between Thresholds 2 and 3 (persistent seed bank). Moreover, the trajectory exhibited by a declining plant species might differ to that exhibited by the same species when it recovers following the removal of a threat. Consideration of the shape of the trajectory of a native species offers insights into the likely level of restoration or management intervention needed to protect the species or to reverse a threshold breach. For example, a high level of alien plant control might be needed before a response can be measured/observed for a native plant species which exhibited a model II shaped decline between Thresholds 1 and 2 (Fig. 1). Similarly, temporal changes in the nature of the threat might alter the shape of the trajectory for a species from one model to another. Such changes might also be observed when a species experiences additional or composite threats (i.e. from additional alien plant species or another type of threat). The three models or different shaped trajectories presented (see Fig. 1) can be used to parameterize population demography models to predict the nature of the population change over time and the likely impact on native species or the likely response of management interventions.

## Plant life-history traits: seed banks and dormancy

Plants possess life-history traits that can make it difficult to state categorically that every last individual has been lost (i.e. extinction), specifically dormant seeds and long-lived seed banks. Many plant species have extremely long-lived seeds and seed banks in the soil (particularly members of the Fabaceae; Pugsley 1928); maximum longevity could be over 400 years (e.g. Ohga 1923). Propagules (seeds) can persist in the soil without any seedlings or adult plants being visible (see Thompson and Grime 1979; Holmes and Cowling 1997*a*; Gooden and French 2014). Furthermore, determining the presence or size of soil-stored seed banks is difficult, partly because they are so spatially heterogeneous (Jones 1998) and/or transient (see Thompson and Grime 1979). For many plant species, it is thus difficult to declare conclusively that no propagules exist; this greatly complicates the accurate pronouncement of plant species as extinct (i.e. at Thresholds 5 and 6). Thus, focusing on whether extinction has occurred in such species ignores the nature of the decline of these species and/or the possibility of the species being ‘functionally extinct’ (i.e. categorical evidence is not available, despite no individuals or propagules being observed, or insufficient individuals occur for the species to survive).

## Alien plant threat prerequisites for demonstrating extinction

We have identified four key elements or prerequisites associated with the nature of the alien plant threat (threatening process) that are needed to demonstrate extinctions. These are (i) the nature of the threat action (i.e. the processes or mechanisms by which an alien plant poses a threat to native plants); (ii) the degree or level to which the threat action is applied (i.e. genes to species-level impact) relative to the tolerance or resilience level exhibited by the native species; (iii) the timeframe over which the threat is active relative to the life history of native plants; and (iv) the spatial relationship between the threat applied in i–iii relative to the distribution of the native plant species (i.e. spatial matching of the threat relative to the risk). We discuss each of these with reference to the six-threshold extinction framework. We believe that understanding the complex interplay between the threat and the species at risk (i.e. as outlined by these four elements) elucidates the prerequisites needed to demonstrate that the threatening process can result in extinction. Examination of these elements reveals that the threat posed by many alien plant species is currently confined to extinction Thresholds 1 and 2. Although the threat posed by some alien plant species may not presently result in the extinction of native plant species (i.e. based on these four elements), this does not mean that we should ignore significant population declines associated with Thresholds 1 and 2, or that with time (prerequisite 3) extinction will not occur.

### Nature of the threat action

The specific processes whereby alien plants contribute to native species declines are well documented (Levine *et al.* 2003). These include direct drivers like (i) competition (Daehler 2003; which includes the effects of density—e.g. Jackson 2005; Gooden *et al.* 2009), excessive resource use (Richardson and van Wilgen 2004) and resource enhancement (e.g. Brooks 2003; Yelenik *et al.* 2004), allelopathy/novel weapons (Ens and French 2008; Inderjit *et al.* 2008), facilitation (Rodriguez 2006) and interference in mutualisms (Reinhart and Callaway 2006; Traveset and Richardson 2014); (ii) disturbance and alterations of disturbance regimes (Mack and D’Antonio 1998; Brooks *et al.* 2004); (iii) habitat transformation (Richardson *et al.* 2000; Asner *et al.* 2008); and (iv) interactions between these drivers (Callaway and Walker 1997). Alien plants also affect native plants indirectly in many ways (Lenz *et al.* 2003; Reinhart and Callaway 2006). While these direct and/or indirect effects illustrate how alien plants contribute to the decline of native plant populations (i.e. through reductions in births, and immigration and increased deaths), linkages with how these effects contribute to native species transitioning across the extinction thresholds have not been explicitly demonstrated. This is partly because the level to which the threat is applied is rarely considered.

### Level of the threat action applied

*Species-level effect*: Although the threat posed by alien plants to native plants can occur at a range of levels (e.g. genetic, individual and population), demonstration of extinction occurs at the *species* level. Such determinations do not, however, take into account whether the species is on a trajectory to extinction, or whether the various components of a species (i.e. genes, individuals or populations) have been affected in ways that facilitate or commit a species to a progression towards extinction (i.e. Thresholds 1–4). Given that the extinction of a species is the culmination of losses that occur at the levels of genes, individuals, and populations (i.e. crossing Thresholds 1–4); leading to a species-level effect (Threshold 5 or 6), there are few studies that examine the effects to native species associated with alien plant invasions at the species level (i.e. gamma-diversity: see measures used below). As outlined below, achieving a species-level effect requires an understanding of the spatial matching of the threat (prerequisite 4).

*Threshold effect*: A growing number of studies from different regions and for many alien plant species show the existence of a threshold effect of alien plant cover or density on native plant species, whereby increased alien plant cover or density decreases native plant species diversity or richness (i.e. Thresholds 1 and 2). For example, Gooden *et al.* (2009) observed a threshold effect of the density of the alien plant *Lantana camara* on native plants in Australia, which differed among groups of native plants (i.e. 30 % *L. camara* cover for ferns, <80 % for herbs and vines, and no apparent threshold for trees and shrubs). McAlpine *et al.* (2015) also found a threshold effect based on the volume of the alien plant *Tradescantia fluminensis* in New Zealand, being around 0.75 m^3^ per 4-m^2^ plot, beyond which there was an abrupt decline in native species richness and species abundance (effects did not differ substantially among different groups of native species). Paterson *et al.* (2011) observed a threshold effect on native plant species richness at <50 % *Pereskia aculeata* density in South Africa, and Coultrap *et al.* (2008) observed a threshold effect on native plant species richness at around 20 % cover of the alien plant *Juniperus occidentalis* var. *occidentalis* in the USA. Also in the USA, Hutchinson and Vankat (1997) found that native tree seedling density was affected by the cover of the invasive vine *Lonicera maackii*, with a threshold of around 15 % cover, above which tree seedling density was consistently <0.5 m^−2^. Species richness of native tree seedlings was inversely related to *L. maackii* cover with a threshold of around 50 % cover. The strongest threshold was for native herb cover which decreased once *L. maackii* cover exceeded 20 %.

Holmes and Cowling (1997*a*) described the sensitivity of native plant species to different categories of invasive stands (uninvaded, recently invaded and long invaded) of *Acacia saligna* in South Africa. They found that in the oldest *A. saligna* stands many groups of native plants were totally absent (Threshold 2), specifically serotinous shrubs (Proteaceae) and that there were fewer ericoid shrubs. The most dramatic decline was observed in the cover of proteoid shrubs in recently invaded stands, suggesting that they may be very sensitive to *A. saligna* invasions. On the contrary, ‘hardy’ successional species like bracken (*Pteridium aquilinum*) and the shrub *Searsia lucida* persisted in long-invaded sites, suggesting that some species have a greater invasion threshold. Yurkonis and Meiners (2004) found that some native plant species are more susceptible to the invasion of *Lonicera japonica* than others in the USA, as a result of reductions in immigration of individual species with increasing *L. japonica* cover (Threshold 1). Although their results illustrate a sensitivity trend of native plants associated with *L. japonica* invasions (Threshold 2), the maximum average cover values observed for *L. japonica* (37 %) were substantially lower than those recorded for thresholds elsewhere (see discussion above), which may be masking the actual sensitivity of native plants. Ogle *et al.* (2000) showed that 24 % of native plant species had been lost (i.e. could not be found again) during a 26-year invasion timeframe of the alien vine *Clematis vitalba* in New Zealand (Threshold 2). Losses of native plant species were not uniform across the various groups of native species. For example, no tall tree species were lost, but 37 % of herbaceous species, 24 % of shrub and small tree species, 21 % of fern species and 9 % of vine species were lost, including populations of at least four threatened species. Gooden and French (2014) reported significant reductions in the species richness of the native plant seed bank and greater increased dissimilarity between the seed bank and the standing vegetation for sites invaded by the alien grass *Stenotaphrum secundatum* in Australia; these results provide evidence for Threshold 3 being breached (i.e. loss of individuals and propagules in the seed bank). These examples show that some groups or species of native plant taxa appear to be very sensitive to the invasion of alien plants and are potentially suppressed or even lost quickly following invasion (i.e. model II for the transition between Thresholds 1 and 2: Fig. 1), whereas others persist with high densities or cover of alien species, which does not preclude the possibility of a model III response in the future (see Fig. 1).

### Timeframe of the threat relative to extinction

There is increasing awareness of significant time lags between the initiation of forcing functions and the response of different components of biodiversity (Essl *et al.* 2015). This clearly applies for plant invasions, where substantial lags between the introduction of an alien plant and any noticeable effects on biodiversity or ecosystems are the norm (Gilbert and Levine 2013). This has led to the discussion of induced extinction debts on native biota, being the time lag between the introduction of an alien species and the extinction of native species (e.g. Byers and Goldwasser 2001; Gilbert and Levine 2013). Importantly, such extinction debt could take several hundred years to manifest (Gilbert and Levine 2013). The short residence time for many invasive plants globally (including many of the poster-child examples of destructive invasive plants) means that the full extent of effects of such invasions on native biota has yet to be manifested; there is likely to be a major extinction debt (see also Richardson and Ricciardi 2013). There is also strong evidence that for some alien plant species the nature of their invasiveness changes over time following the initial invasion (subsequent spread is contingent upon plastic responses or genetic adaptation to the new environment) (Dietz and Edwards 2006). This may in turn result in changes in their impacts upon native species, highlighting that the effects are dynamic, not static. This is not to say that decreased effects over time have not been documented (Dostál *et al.* 2013; Sulivan 2014).

Gilbert and Levine (2013) showed that extinction debts attributed to alien grass invasions are the result of two main processes: (i) a decrease in the size of native refugia and (ii) a decline in the dispersal ability/capacity between refugia (as observed elsewhere; e.g. Gooden and French 2014). Hylander and Ehrlén (2013) argue that extinction debts arise because (i) individuals may survive in resistant life-cycle stages long after habitat quality changes (e.g. as propagules in the seed bank following an invasion—Thresholds 2–4); (ii) extinctions of small or declining populations due to stochastic events are not immediate (i.e. such events may only be triggered every 50 years—Thresholds 2–4); and (iii) individual populations may survive long after dispersal between populations has ceased (Thresholds 2 and 3). Moreover, the outcomes of such events are dependent on time, scale and the degree of habitat specificity exhibited by a species (Cousins and Vanhoenacker 2011).

*Evidence for extinction debts***:** Although not explicitly elucidated as such, there is much evidence of extinction debts in the literature on alien plant invasions. For example, in South Africa, many native fynbos species in sites invaded by *Acacia saligna* had smaller soil-stored seed banks, suggesting the potential for future losses (Holmes and Cowling 1997*b*). Miller and Gorchov (2004) document reduced seed production in three native perennial herbs in invaded *Lonicera maackii* stands in the USA, suggesting that although there was no reduction in survival, the long-term effects of reduced recruitment may lead to an extinction debt. In Australia, Gooden and French (2014) described several indicators of extinction debt following the invasion of the alien grass *Stenotaphrum secundatum*, including significant reductions in the native plant species richness of the seed bank (driven by reduced germinant density following invasion), increased dissimilarity between seed banks and standing vegetation and recruitment limitation (specifically species losses were observed for herbs, graminoids and vertebrate-dispersed native plant species). Ogle *et al.* (2000) observed that populations of at least four threatened plant species could not be found following the invasion of the alien vine *Clematis vitalba* in New Zealand. These examples illustrate that the effects of alien plants can propel native plants across Thresholds 1–3 on the extinction trajectory, thereby elevating their risk of extinction.

### Spatial matching of the threat relative to the risk

**Figure 2.**
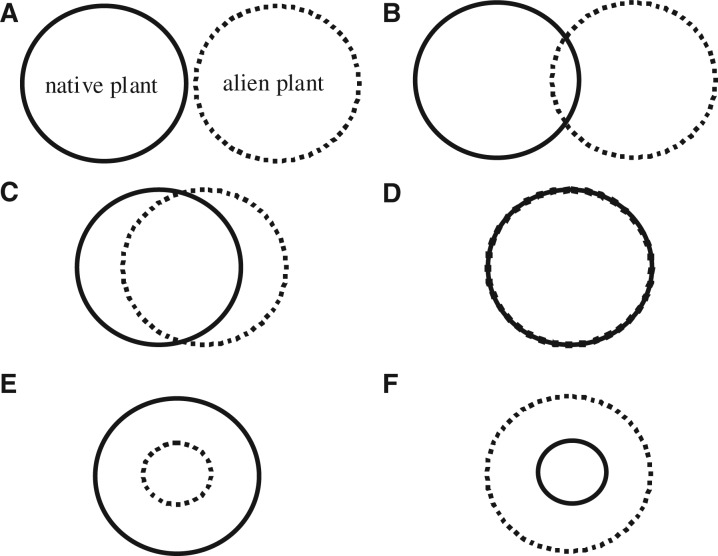
Example of the distribution of an alien plant (dashed line) relative to the distribution of a native plant (solid line), in which (A) there is no overlap in their distributions; (B) there is partial overlap on the margins of their respective distributional limits; (C) major overlap including core distributions of each species; (D) there is complete overlap; (E) the distribution of the alien is a subset of the native's distribution; and (F) the distribution of the native is a subset of the alien's.

**Figure 3.**
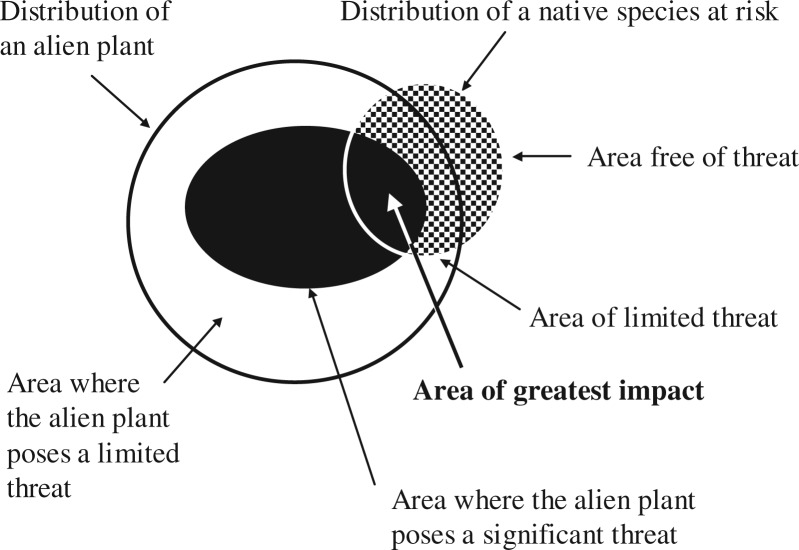
An example showing the spatial relationship and nature of the threat between the ranges of an alien and a native plant (reproduced from Downey 2010, from *Invasive Plant Science and Management*, with the permission of Allen Press Publishing Services).

Demonstrating that extinction could occur is intrinsically linked to the degree of spatial matching of the threat (i.e. the spatial distribution of the alien plant) relative to the risk (i.e. the distribution of the native species under threat). Extinction can only be demonstrated in instances where the threat is applied across the entire distribution of the native species over a sufficient period for the extinction to occur (see prerequisite 3 above). Although some authors have acknowledged that different effects from alien plants occur at different spatial scales (e.g. Lawes and Grice 2010; Powell *et al.* 2011; Rejmánek and Stohlgren 2015), such examples do not consider the degree of distributional overlap or spatial matching, although Gilbert and Levine (2013) do outline a declining relationship between the proportion of the habitat lost and the persistence of native species. Such assessments are needed, but are rarely undertaken (Downey 2010). We consider six theoretical examples to illustrate the range of potential overlaps (Fig. 2A–F) and how each can determine the type of outcome that might occur to native species relative to the six extinction trajectory thresholds (including demonstrating extinction). By combining these examples with the approach outlined by Downey (2010) to understand the spatial relationship of alien plants and the threat to native plants (i.e. Steps 3 and 4), a more comprehensive framework can be produced. Although example (a) (Fig. 2A) shows no direct effect, indirect effects cannot be ignored (e.g. Williams and Baruch 2000; D’Antonio and Hobbie 2005). For an alien plant species to pose a species-level effect to a native species (i.e. potential extinction—Thresholds 5 and 6), the level of spatial matching must represent either example (d) or (f). Neither of these will necessarily lead to the extinction of native plant species as the spatial nature of the effects of an alien plant are unlikely to be uniform across their entire distribution (Fig. 3; also see Pouteau *et al.* 2015); the smaller the distributional subset of native plants, the greater the likely risk.

In examples (b) and (c) (Fig. 2B-C), potential effects of alien plants on native plants are restricted to individual or population levels (Thresholds 1–3). Although individuals and populations of the native plant may become extinct due to the effects of an alien plant (i.e. local extinctions), a species-level effect attributable solely to the alien plant species is not possible because part of its range is in areas not affected by the invasion (this could be due to biogeographic variations within the species distribution, or biogeographic barriers preventing complete matching). However, this may change over time (prerequisite 3 see above), especially for alien species which have yet to reach their full distribution, either because of dispersal limitation or because of changes to their realized niches (see Lawes and Grice 2010) or because they are still expanding; such temporal changes have led to conflicts in evidence (Dietz and Edwards 2006). Moreover, the likelihood that such effects are uniformly applied decreases as the distribution size increases. This is because the effects are likely to be mediated by other factors such as climate, topography and biogeographical variations. Inevitably, relationships between species fitness and the niche overlap between the invasive and native species will determine the outcome of their interaction (see MacDougall *et al.* 2009; Rejmánek 2011). Thus, the probability that an alien plant will invade the entire range of a native plant species that is widespread is low; therefore, the ability of an alien plant species to pose a species-level impact (Thresholds 5 and 6), at least in the short-term, is also low. This is not to say that population-level extinctions (i.e. Thresholds 2–4) are not important (see Ricciardi 2004).

## Evidence for extinctions in plants—are we collecting appropriate data?

Demonstrating that a species is categorically extinct is difficult; this is further complicated because in many instances appropriate data are not collected over sufficiently long periods to demonstrate conclusively that extinctions have occurred. We identify and discuss a range of data deficiencies that contribute directly to our ability to demonstrate categorically that extinction has occurred as a result of alien plant invasions. These are (A) a poor understanding of many alien plant species and their threat/impact to native plants, (B) the data presented cannot demonstrate extinctions and (C) lack of meta-analyses or global datasets of the native species at risk. Thus the current lack of evidence for extinctions associated with not collecting the appropriate date should not be misconstrued as indicating that there is no effect (i.e. Type II error).

### Many alien plants are poorly studied, especially in terms of their potential impacts

*Impacts from alien plants*: Impacts associated with most invasive alien plants have not been studied or are poorly understood or documented. For such alien species the threat or extinction risk they pose to native plant species is unknown. In fact, most studies on impacts of invasive plant species on native species have examined a relatively small number of alien species. For example Vilà *et al.* (2011) documented only 135 taxa in a global review of impacts. Although the effects of many alien plants on native plants have not been examined, results for those that have been studied provide strong evidence for breaches of Thresholds 1 and 2 and, to a lesser extent, Threshold 3. Moreover, the combined effect of multiple invasive plants is rarely studied or considered (see below).

**Figure 4.**
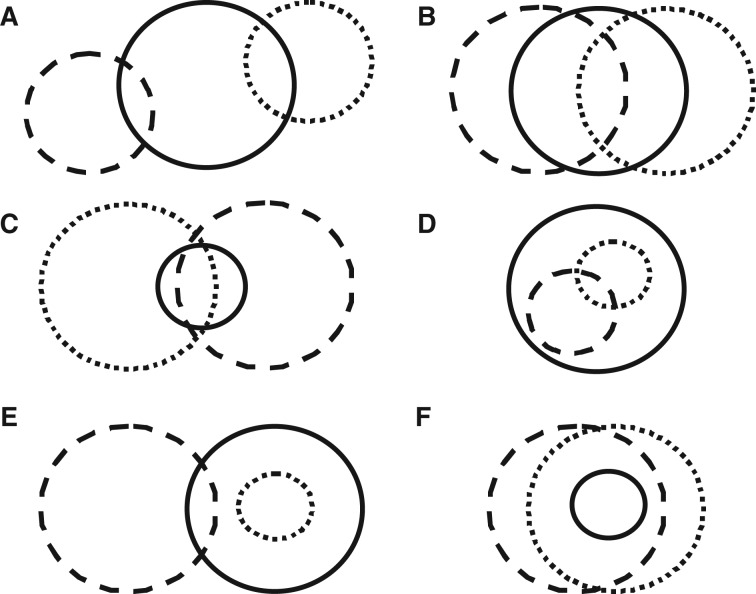
Example of the distribution of multiple alien plant species (dashed and dotted lines) relative to the distribution of a native plant (solid line), in which (A) there is partial overlap on the margins of their respective distributional limits; (B) major overlap including core distribution of the native species; (C) there is complete overlap; (D) the distribution of both alien plants is a subset of that of the native; (E) the distribution of one alien is a subset of the native's and the other alien has partial distribution overlap; and (F) the distribution of the native is a subset of that of both the alien plant species. Note: the complete overlap of individual alien plant species has not been presented.

*Effects of multiple invasive alien plants*: Apart from discussions around instances of ‘invasional meltdown’ (*sensu* Simberloff and von Holle 1999), many of the studies of the effects of alien plants on native species have typically taken a single-species approach (Downey and Grice 2008), a notable exception being the study by Pearson *et al.* (2015). Many authors have, however, outlined how secondary alien plant species become problematic after management (e.g. Zavaleta 2002; Holmes *et al.* 2008; Reid *et al.* 2009) and that the effects from multiple alien plant species can be cumulative (Adair and Groves 1998; Lawes and Grice 2010). Coutts-Smith and Downey (2006) found that 43 % (*n* = 88) of native plant species threatened by invasive plants were threatened by more than one alien plant species, with the maximum being more than 10. Thus, the combination or compound outcomes of such effects may be more important for the long-term survival of native species than those from a single invasive plant species. However, such compound effects have rarely been considered, let alone measured, in determining native species declines or extinctions, again with a notable exception being the study of Pearson *et al.* (2015). We use a series of examples to show how the spatial relationship from multiple alien plants may affect native species differently (Fig. 4). Considering the effects of individual invasive plant species in isolation may mask any effects from multiple alien plants, especially if such effects occur in different parts of a native species distribution (Fig. 4A–F), have differing lags or are studied independently. Also see the discussion pertaining to Fig. 3. Although the effects of one invasive plant species may lead to breaches of Thresholds 1–3, the combined effects of multiple alien plants may result in breaches of Thresholds 4–6, based on the cumulative effect—something that will not emerge from studies of single invasive alien plant species.

*Impacts on native plants*: The potential impacts of invasive alien plants on native plant species are extremely varied, and have been measured in many different ways, allowing many different conclusions to be drawn regarding the magnitude and consequences of the effects (Kumschick *et al.* 2015). Moreover, quantification of the decline of native species attributable to alien plants has only recently been initiated in many parts of the world (e.g. Gaertner *et al.* 2009). The major limitation to inferring extinctions is not the models that are used, but the lack of appropriate data required to parameterize such models (Boakes *et al.* 2015).

An additional complication is that for many native species at risk, the threat from alien plants is only documented in generic terms (i.e. specific alien plant species are rarely identified or described). For example, Coutts-Smith and Downey (2006) found that for almost half of the 419 threatened species affected by alien plants in New South Wales (NSW), no specific alien plant species could be identified (i.e. the threat was described generically as being caused by ‘weeds’ for example). Furthermore examination of the IUCN Red List database (IUCN 2015) revealed that of the 482 plant species that listed alien plants as a threat, 59 % contained similar generic threat descriptions. Information pertaining to a specific alien plant species was thus available for fewer than 40 % of species (Table 1). For many native species, the risk posed by alien plants has not been properly documented. It is, therefore, difficult to determine whether native plant species are being driven to extinction from alien plants if no one records the native species affected by such alien species or the alien species that pose the threat.

**Table 1.** The population trend of IUCN Red-listed plant species threatened by alien plant species between the current and previous assessments (data extracted from IUCN 2015).

Population trend for plant species threatened by alien plant species	IUCN Red List categories^a^					
	Critically endangered (*n*)	Endangered (*n*)		Vulnerable (*n*)	Near threatened (*n*)	Least concern (*n*)
Increasing	2 (0)	0 (2)		3 (0)	1 (0)	0 (1)
Decreasing	28 (36)	26 (32)		13 (18)	5 (15)	6 (4)
Stable	5 (6)	4 (2)		4 (6)	2 (2)	16 (21)
Unknown	17 (19)	10 (12)		11 (5)	13 (5)	15 (23)
No details^b^	3 (44)	1 (16)		5 (15)	0 (0)	6 (2)
Total number of plant species	55 (105)	41 (64)		36 (44)	21 (22)	43 (51)
Grand total across all categories			196 (286)			

^a^In August 2015 all plant species listed on the IUCN Red List threatened by alien species were examined to determine those threatened by alien plants and those for which a specific alien plant species was identified as posing a threat. The data were then separated into two categories: those with a specific alien plant threat, and those with a generic threat from alien plants (i.e. weeds, exotic plants, etc); in brackets. All other alien species were removed along with species threatened by a generic threat from alien species.

^b^Entries were blank in the IUCN database.

In many instances, however, the most sensitive species may be lost long before any studies are undertaken and thus their effects may be undocumented. This means that there is a potential sampling bias in determining the extinction risk for the species mostly likely to be at risk.

### Data presented cannot demonstrate extinctions

*Population dynamics*: The extinction of a species involves factors that affect the four components of the population dynamics of a species: i.e. fecundity [birth—seed production], death; immigration and emigration [dispersal in plants]. There is growing evidence from many studies across multiple regions that alien plants negatively affect all these components individually. For example, many alien plants (i) reduce seed production rates (e.g. Miller and Gorchov 2004); (ii) increase mortality rates (e.g. Gorchov and Trisel 2003); (iii) reduce immigration rates (e.g. Yurkonis and Meiners 2004); and (iv) although reducing emigration rates may have a positive benefit for the dynamics of a specific population, such reductions translate to reductions in immigration rates to other populations of a native plant species. For example, Gilbert and Levine (2013) outline how alien plants can reduce dispersal between refugia of native plant species, which encompasses both immigration and emigration between such refugia. Unfortunately, we know of no studies that have explored such effects of alien plants for all four components collectively for a single native plant species either across the species distribution or for a specific population.

The loss of multiple populations of a species across its entire range may be of significant concern for the extinction risk of a species (Hobbs and Mooney 1998), without resulting in its extinction (Ricciardi 2004) (Threshold 3). Given that thousands of individuals (not hundreds) of a species are needed for populations to have a chance of overcoming or withstanding a major threat (see Traill *et al.* 2010), any major decline in the number of individuals of a species could be important for the species overall survival.

*Measures used*: Many alien plant studies are not designed to assess whether extinctions have occurred (i.e. Thresholds 4–6). For example, Yurkonis *et al.* (2005) argue that using changes in species richness to assess the impacts of alien plants on native species will not adequately predict or describe the effects of invasion (or provide evidence for Thresholds 1–3). To illustrate this point, Yurkonis and Meiners (2004) measured the net change in species richness following alien plant invasions in which extinction is partly offset by colonisation. It is the composition of species present and the degree of similarity, not simply the number of species present, that are important. Unfortunately many studies that examine the effects of alien plants on native species use measures of species richness (see Holmes and Cowling 1997*a*; Gooden *et al.* 2009). Furthermore, collective species measures (i.e. species richness) could potentially mask losses of some species in instances where additional species are also recorded (i.e. the losses are off-set by additions). Although diversity measures and assessments of evenness can provide information on changes in the density of species, they are rarely used to determine the effects of alien plants on native plants and are unlikely to demonstrate that every individual of a species has been lost.

Most studies that have explored the effects of alien plants on native plants have looked at alpha-diversity (i.e. within a habitat), and not beta-diversity (i.e. between habitats) or gamma-diversity (i.e. within a region). This makes it almost impossible to document even population-level effects (i.e. Thresholds 1 and 2—also see discussion above), let alone effects at the species level (i.e. Thresholds 3–6). For example, studies of species richness or density in a particular invaded site or invaded region, compared with uninvaded sites which show a decline in species richness in invaded areas (e.g. Holmes and Cowling 1997*a*; Gooden *et al.* 2009), can identify declines of individuals or populations, but not the extinction of a species.

Genetic losses can occur from the loss of individuals and their subsequent genetic diversity, or more broadly from the loss of multiple individuals and/or populations (i.e. leading to a reduced population size), and the subsequent reduction in gene flow and transfer (Essl *et al.* 2015) (Thresholds 1 and 2). Losses of individuals of a species are rarely recorded because many studies only assess species richness (e.g. Jackson 2005; Srinivasan *et al.* 2007; Stohlgren and Rejmánek 2014), rather than tracking the fate of individuals over time). Studies that document individual losses typically refer to such losses as reduced survival (e.g. Greene and Blossey 2012) and not the loss of individuals in the context of an extinction trajectory (Threshold 1), partly because other individuals and/or populations of the species are not affected and individuals might be replaced through recruitment (births) and immigration. Also, the death of individual plants can take a very long time, especially in long-lived species like trees (see Cordell and Sandquist 2008), despite the presence of the threat. Furthermore, while individuals may not survive or recover from a threatening process, populations and species are inherently more resilient; this could mask observations of the crossing of Threshold 1. While clear evidence for losses of populations of a species (i.e. a localised extinction of a species or extirpation: Threshold 2) is uncommon in the literature (a notable exception being Ogle *et al.* 2000), many studies present data to infer potential future losses, for example increased dissimilarity between seed bank and standing vegetation combined with reductions in seed bank species richness (e.g. Gooden and French 2014) or reductions in the frequency of species between invaded and uninvaded sites (Holmes and Cowling 1997*a*; Yurkonis *et al.* 2005). Such declines are rarely considered in the context of the extinction trajectory of the species (i.e. Threshold 2).

More broadly with respect to determining extinctions, Possingham *et al.* (2002) argue that measures of species richness or diversity are not appropriate, but that the focus needs to be on the fate of individuals. This point is also made by Boakes *et al.* (2015) who emphasize that a major limitation in demonstrating extinctions is the lack of data demonstrating conclusively that no individuals of a specific species exist.

*Seed banks*: Because seed banks are not routinely sampled, marked declines in native plant species with persistent seed banks in invaded communities may be underappreciated (D’Antonio and Meyerson 2002), despite seed bank size being a critical factor in population dynamics and determining extinctions. Such declines could be caused by reduced seed input through interference of the invasive species with various aspects of reproduction of the native species (Traveset and Richardson 2006); even highly persistent seed banks decay rapidly once seed input has been reduced or stopped (Thompson and Grime 1979). Consequently, the absence of native species in the above-ground vegetation in long-invaded areas, while conforming to a Threshold 3 breach, is usually a portent of their imminent local extinction (Holmes and Cowling 1997*a*) which represents Threshold 4.

*Sources of evidence***:** Many different types of data and sources of evidence have been used to describe and document the effects of alien plant species on native species. The importance of some data types has in some instances been overstated or misconstrued which may have resulted in misleading conclusions (Rejmánek 2012). For example, in many instances, assessments of threats (e.g. Wilcove *et al.* 1998; Coutts-Smith and Downey 2006) have been misconstrued as assessments of actual impacts which could lead to the spurious conclusion that the problem has been overstated. Clearly, caution is required when using the different types/sources of data used to draw conclusions. To prevent such problems we identify ten commonly used categories of data that have been used to document the effects of alien plant species on native species and describe what information can be reliably derived from each with respect to the six extinction thresholds (Table 2). Although there may be a dearth of scientific studies documenting extinctions (Thresholds 5 and 6), numerous studies have documented negative impacts (specifically relating to Thresholds 1–3), and substantial information is available (which is increasing with time) from the other types of data that alludes to underlying trends (Thresholds 1 and 2: also see Table 3) that show that many native plant species are transitioning across these thresholds as a result of alien plant invasions. Such insights cannot be ignored simply because they are not derived from robust scientific studies such as those advocated by Barney *et al.* (2015) and Kumschick *et al.* (2015). The collective trends derived from the different information sources show that many native plants species are likely to be on an extinction trajectory (i.e. crossing Thresholds 1 and 2).

**Table 2.** Ten categories of data commonly used (see table footnote for details) to describe the effects of alien plants on native plant species. The type of information that can be reliably derived from each data category is listed and the respective extinction trajectory threshold (see text for details).

Data categories^a^	The type of information that can be reliably derived^b^	Extinction trajectory threshold^c^	Reference
Observational or anecdotal notes	Provides an indication of a potential threat	1 and 2	1, 2
Qualified observations^d^	Provides an indication of a potential threat	1 and 2	1, 2
Documented trends^e^	Documents a potential threat supported by rudimentary data or information	1 (potentially 2)	3
Cited unpublished results	Provides an indication of a potential threat	1 and 2	3
Expert assessments	Describes the nature of the threat	1 and 2	3
Determinations	Assessments based on available data, showing evidence of declines over time and the level or nature of the decline, typically based on set criteria (see Mace and Lande 1991). Extinctions can be documented	1 and 2 (potentially 3)	4
Mixed data compilations	Assessments of the available information from multiple sources and types of data, which documents the broader nature of the threat across multiple species and the likely consequences (impacts). Such studies can be replicated over time to illustrate changes in the trend	1 and 2 (potentially 3)	5, 6, 7
Prioritisation models	Provides the justification for management actions, the outcomes of which need to be monitored	1 and 2 (potentially 3)	2, 3
Scientific studies	Studies that describe and document evidence of impacts and the nature of that impact. Note: there is huge variability in the type of data derived here. Extinctions can be documented	1–6	8, 9, 10, 11
Meta-analyses of such studies	Compilations of available data (typically from scientific studies) that provides a broader assessment of the impacts and the likely consequences	1–6	12, 13

References: 1. Downey (2006); 2. Turner and Downey (2010); 3. Downey (2010); 4. IUCN (2015); 5. Adair and Groves (1998); 6. Wilcove et al. (1998); 7. Coutts-Smith and Downey (2006); 8. Miller and Gorchov (2004); 9. Gooden et al. (2009); 10. Greene and Blossey (2012); 11. McAlpine et al. (2015); 12. Gaertner et al. (2009); and 13. Vilà et al. (2011).

^a^Ten commonly used data categories derived from reviewing published information on alien plant threats to native plants species—the different sources (i.e. data types/information) used were compiled and then grouped into 10 categories that were described using words to best represent the collective source for each group.

^b^Definitions of *Threat* and *Impact* as described by Downey et al. (2010).

^c^The six extinction trajectory threshold numbers (see text for details and Fig. 1).

^d^Observational data (i.e. based on a degree of systematic assessments over time, or from observations derived from the outcomes of management actions) which may or may not be published.

^e^Non-scientific results (e.g. data derived from photo points illustrating a ‘change’).

**Table 3.** The number of native plant species formally as listed threatened (i.e. under IUCN Red List or threatened species legislation) for which alien plants are described as one of their threats.

	The number of threatened plant species that are threatened by alien plants in each threat category				
Country	Threatened (total)	Critically endangered	Endangered	Vulnerable	Reference
World (ICUN Red List)	196^a^	55	41	36	1
South Africa	1426	239	504	683	2
USA	602				3
Australia (NSW)	279		166	113	4
Australia (Victoria)	16				5
Australia (National)	57		57		6
New Zealand	103^b^		26	33	7
Tahiti	32	15	2	15	8
Mauritius	>4^c^	>2^c^	>2^c^		9

References: 1. IUCN (2015); 2. SANBI (2015); 3. Wilcove et al. (1998); 4. Coutts-Smith and Downey (2006); 5. Adair and Groves (1998); 6. Leigh and Briggs (1992); 7. Reid (1998); 8. Meyer and Florence (1996); 9. Baider and Florens (2011).

^a^see Table 1—includes all threatened categories and only species for which a specific alien plant was identified. A further 286 plants are threatened by a generic listing of alien plants.

^b^Includes 33 plant species listed as rare.

^c^The authors outline two species which were presumed extinct that recovered following alien plant control as well as ‘several other’ Critically Endangered and Endangered plant species—the exact number was not provided.

There are multiple assessments of the threat posed by alien plants to native plants formally listed as threatened (i.e. under the IUCN Red list). These reveal that over 2000 native plant species globally are under threat from alien plants (Table 3). For example, Coutts-Smith and Downey (2006) documented that almost 60 % of the native plant species listed as Endangered (166 of 279 native plant species) and 40 % listed as Vulnerable (113 of 279) in NSW, were threatened by alien plants, which together represented 49 % (279 of 565) of all listed native plant species in this state. These numbers are likely to be even higher, as Coutts-Smith and Downey (2006) also documented 64 ecological communities that were threatened by alien plant species. In New Zealand, 72 % of the threatened plant species with the highest priority for conservation were threatened by alien plant species (Molley and Davis 1994), highlighting the significant impact alien plant species pose to such native plant species.

There are also a few studies that have documented the extent of the threat posed by specific alien plants to native plants. For example, Turner and Downey (2010) assessed the threat posed by the alien plant *Lantana camara* to native plants in Australia and produced a list of 1321 native plants at threat because of its invasion. The authors subsequently assessed these native plants to determine which were most likely to change to a higher threat status (i.e. progression along the extinction trajectory) if *L. camara* is not controlled soon. This revealed 275 native plants in the highest category (i.e. those for which extinction is highly likely). In another study, Downey (2010) described the threat posed by the alien plant *Chrysanthemoides monilifera* to 157 native plant species in NSW, of which 19 were assessed as being at the greatest threat.

*Timeframes*: Based on the definition of extinction (see above), the time required to collect data to determine that any native plant species has been driven to extinction from an alien plant exceeds that of most of the very few long-term studies in invasion ecology (e.g. Downey and Smith 2000), virtually none of which span more than 50 years. The problem is compounded because very few species (either native or alien) are monitored systematically over sufficiently long periods.

Habitat fragmentation studies provide useful insights on the value of long-term data in that there is strong evidence that native species extinction rates are an artefact of exposure time (i.e. time since fragmentation); the longer patches have been fragmented, the greater the extinction rate (Ferraz *et al.* 2003). With respect to invasion, Lawes and Grice (2010) describe how temporal and spatial scales play important roles in our understanding of the outcomes. The absence of long-term data, therefore, seriously hampers our understanding and ability to state categorically that extinctions have occurred.

### Lack of a global database of native plant species affected by alien plants

To determine extinctions, extinction rates, extinction trajectories or the six thresholds outlined here, information on specific native plant species affected by alien plant species and the level of such effects are needed. However, such information is currently scarce for many native plant species (see above). This is despite the fact that many studies publish lists of native plant species that are present in uninvaded sites but absent from invaded sites (e.g. Holmes and Cowling 1997*a*; Ogle *et al.* 2000; Miller and Gorchov 2004; Srinivasan *et al.* 2007; Gooden *et al.* 2009; Sharma and Raghubanshi 2011; Gooden and French 2014; McAlpine *et al.* 2015) (Threshold 2). There are also many studies that detail the response of native species to alien plant control (i.e. evidence that the threshold is not irreversible). Unfortunately, there are many more studies that only show the ‘collective’ result (i.e. a decline in species richness) without documenting any specific native plant species actually affected (e.g. Yurkonis and Meiners 2004). This is in part because the emphasis of many studies is on the alien plant species, despite the fact that the outcome is the effect to the native plant species.

Although many studies have been done on multiple alien plant species from many different countries, very few attempts have been made to collate information on specific native species that are effected by alien plants (i.e. across studies) and we know of no studies that have examined the effects on a native plant species across its range, despite such data being critical for determining the role of alien plants in native species declines (Thresholds 3 and 4) and their extinction (Thresholds 5 and 6) (also see text above).

## Alien plant management and restoration of invaded sites

Major efforts have been made globally to control alien plant species and restore invaded habitats over many decades (see Hobbs and Mooney 1993; D’Antonio and Meyerson 2002; Beater *et al.* 2008; van Wilgen *et al.* 2011), and many of the ‘worst’ invasive alien plant species are or have been the target for active management and restoration. The benefits of such interventions in terms of preventing extinctions of native plant species, although relatively undocumented, may well have prevented extinctions given the large number of species currently threatened (see Table 3). Such measures have undoubtedly prevented or delayed partial extinctions (i.e. reversing breaches of Thresholds 1–3) for many plant species, given that many management programs have led to important conservations outcomes worldwide (Zavaleta 2002). Such control and restoration efforts may have masked or offset potential extinctions. Thus such ‘species credits’ (i.e. where otherwise ‘doomed’ species are likely to benefit from restoration efforts: see Hanski 2000) and reversals of breaches of extinction trajectory thresholds must be considered in the extinction debate.

*Measuring the response*: Although Reid *et al.* (2009) found that the control of alien plants did not necessarily result in native species recovery, the basis for such results needs to be considered. For example, most of the studies examined by these authors measured species richness, which could mask any detrimental trends (see above). Also, the control rarely targeted sites where the probability of achieving a conservation outcome was high (as described by Downey 2010; Downey *et al.* 2010). Furthermore, the attempts to remove alien plants can have a range of negative impacts on native species (Ogle *et al.* 2000; D’Antonio and Meyerson 2002; Coutts-Smith and Downey 2006; Beater *et al.* 2008; Skurski *et al.* 2013), and as Flory and Clay (2009) found, different alien plant control methods led to different responses by native species. Many alien plant management programs also fail to account for the effects of multiple alien species. Lastly, many studies fail to implement appropriate monitoring protocols to determine or demonstrate the conservation outcomes (Blossey 1999; Downey 2010, 2011). Many studies have, however, shown positive effects on native species following alien plant control (Carlson and Gorchov 2004; Hartman and McCarthy 2004; Andreu and Vilá 2011).

*Misplaced focus of management outcomes*: Many management programs for alien plants have historically focused exclusively on removal of alien plants, which is unlikely to lead to successful outcomes in many situations (see Hobbs and Humphries 1995). Less emphasis has been placed on understanding and measuring the outcomes of such control actions in terms of native plant species protection and recovery (Downey 2011), although there have been advances in this regard in recent years (e.g. Randall *et al.* 2008; Downey 2010; Downey *et al.* 2010; Gaertner *et al.* 2012). Thus the lack of focus on conservation outcomes from alien plant management has directly contributed to a lack of data on the response of native species to control and restoration efforts (e.g. Luken 1997; Downey 2010).

Given that many land managers identify the desirable system changes they hope to achieve (Hobbs and Mooney 1993; Luken 1997) (i.e. a reduction in the negative effects of alien plant species to native species—Thresholds 1 and 2), their management actions [control and restoration] should aim to prevent the worst-case scenario (i.e. extinctions), and thus just because they have not measured their actions appropriately does not translate to an absence of evidence for prevention of extinctions (i.e. Type II error). Arguing against alien plant management actions (e.g. Sagoff 2005), based on a type II error is unwarranted, especially as processes are now in place in many countries to focus limited conservation resources on areas where alien plant management is likely to result in the greatest conservation outcome (i.e. Downey *et al.* 2010), and ignores the positive outcomes of reversing threshold breaches in protecting native species in the long term.

*Positive outcomes from management*: There are many studies that show positive conservation outcomes from alien plant management (i.e. Barton *et al.* 2007; Flory and Clay 2009; Baider and Florens 2011; Meyer *et al.* 2012), and that the threat from alien plants to high conservation areas (i.e. biodiversity hotspot) is both significant and great (Stohlgren *et al.* 2003). This highlights the need for active intervention to protect native species and the evidence for reversals of threshold breaches. Although the data presented in studies of such actions rarely assesses the recovery of the native species present, there are many examples from other data sources (as outlined in Table 2) that show native species recovering following alien species control.

Alien plant control actions can clearly alter the extinction trajectory and reverse threshold breaches of native plants. For example, Meyer *et al.* (2012) showed that control of the invasive alien tree *Miconia calvescens* on the island of Tahiti increased native plant species richness at all sites and that a rare plant species was able to establish post-control. The successful biological control of mistflower (*Ageratina riparia*) in New Zealand reduced the risk of extinction for two threatened native plant species (Barton *et al.* 2007). On Mauritius, Baider and Florens (2011) found that alien plant control led to the recovery of several endangered species, including two plant species that were presumed to be extinct (neither of which had been recorded for > 50 years). Such examples demonstrate that if nothing had been done to manage invasive plants, their overall impact on native plant species would have been more severe than what is currently documented. By the time, that there is universal agreement that invasive alien plants are contributing substantially to the extinction risk for many native plants, the invaders are likely to be established at high densities over very large areas, making protection of native species very difficult (see Byers and Goldwasser 2001).

## The framework

The framework presented here proposes a refocusing of the debate regarding the impact of alien plant invasions on native plant species onto six thresholds along a species extinction trajectory, of which extinction is the last threshold or end point (Fig. 1). This approach shows that concentrating only on the end point (i.e. Thresholds 5 and 6) provides an inappropriate foundation for assessments and/or for considering changes in the position of native plant species on the extinction trajectory (i.e. Thresholds 1–4).

Active management of alien plants and restoration of invaded sites has potentially off-set or delayed some extinctions (i.e. by slowing progression along the trajectory) or enabled some threshold breaches to be reversed. Such affects are likely to have been masked by only focusing on the end point (i.e. Thresholds 5 and 6); positive benefits to native species can, therefore, easily be misconstrued as the absence of the end point.

## Conclusions

### Comparisons between extinction rates and processes for alien plant and animal species are unhelpful

Some authors have compared the high extinction rates associated with alien animal invasions and the extremely low rates associated with alien plant invasions (see data presented by Sax and Gaines 2008) and have questioned the importance of alien plant invasions as a threat to global biodiversity. We argue that such arguments are not helpful, and indeed seriously misleading, given the six thresholds outlined here. Animal species are generally much more susceptible to rapid extinction than are plants. Demonstrating extinctions is also much more straightforward for most animals than for plants, since most animals lack dormant propagule banks. Alien animal predators (the main contributor to animal extinctions; see Dickman 1996; Coutts-Smith *et al.* 2007; Salo *et al.* 2007) are highly mobile and can actively search for native animals and are thus able to inflict rapid population- and species-level effects. The impact of alien animal predators on native species is usually direct (the quick death of individuals) whereas the death of individual native plants due to the presence of alien plants is typically much more prolonged. Predator–prey models show that predator growth is intrinsically linked with prey density (e.g. the functional response: see Carlsson *et al.* 2010)—this is not the case with the interactions between alien and native plants. Thus, comparing extinction rates between animals and plants is not helpful.

### Alien plants and other threatening processes

Although there are no conclusive examples that document the extinction of native plant species solely as a result of alien plant invasions, five IUCN Red-listed extinct plant species have alien plants listed as one of the causes of their extinction (IUCN 2015). Thus we need to consider the effects of invasive plants in conjunction with other threatening processes, as it is rare that one threatening process in isolation leads to the extinction of a species. Further examination of the 196 species on the IUCN Red List database (IUCN 2015) threatened by alien plants (see above and Table 1) revealed that 53 % (104 species) contained information that alien plants were one of the main threats to the species; this trend was fairly evenly distributed across all IUCN categories. No threat other than from alien plants was described for 9 % (*n* = 17) of these plant species. In addition, there is evidence that alien plants can contribute to the further decline of native species previously affected by other threatening processes (see Van der Wal *et al.* 2008).

### IUCN categories and extinction trajectory thresholds

Although not explicitly linked to the thresholds identified here, the IUCN threat categories (i.e. vulnerable and endangered) also represent points along the extinction trajectory and can be used to show changes in the threat status over time. Examination of the IUCN Red List population trend data for plant species threatened by alien plant species (IUCN 2015) shows that most plant species threatened by alien plants are in decline across all threat categories (Table 1). Very few are improving in status (i.e. becoming less susceptible to extinction).

### The framework in context

Although there has been much debate about whether alien plants lead to native species extinctions, the discussion has not addressed the underlying evidence for extinctions (a notable exception being Rejmánek 2012) or examined whether native plant species are on trajectories to extinction (or consider thresholds along such a trajectory). Instead the end-point or threshold (i.e. extinction) has been the sole focus. Our framework illustrates the importance of the extinction trajectory and that other key thresholds along the trajectory are also important in determining the effects of alien plants on native plants. Our review also highlights a range of threat prerequisites needed to demonstrate extinction, the types of data needed to determine extinctions, timeframes needed to demonstrate extinctions and how active management of alien plant species is likely to have off-set many extinctions and/or altered the extinction trajectory for many others. All these factors must be considered when assessing the potential threat to native plant species from alien plants at all the six thresholds outlined. The six-extinction trajectory threshold framework provides guidance for future work around understanding and assessing impacts.

It is the direction of change that is fundamentally important (i.e. the extinction trajectory and the thresholds that have been breached along the trajectory), not whether a native plant species has actually been documented as going extinct due to an alien plant species based on a snapshot view. Furthermore, managers cannot intervene once a species goes extinct, but they can intervene to stop extinctions or to reverse breaches of thresholds along the extinction trajectory, if they know species are on an extinction trajectory, which is the basis for developing the IUCN Red List and the associated threat categories. We have provided both a framework and sufficient evidence to demonstrate that many native plants species are currently undergoing a negative directional change (i.e. they have breached thresholds along the extinction trajectory) as a result of alien plant invasions. For many species, this requires immediate action to prevent future declines and likely extinctions. Much work remains to be done to derive practical ecosystem-and taxon-appropriate metrics for assigning impact of invasive plant species on native plant species. The approaches advocated by Barney *et al.* (2015) and Kumschick *et al.* (2015) are steps in the right direction, as is the scheme proposed by Blackburn *et al.* (2014) for classifying impacts of invasive species based on objective criteria for assessing multiple impact mechanisms. Improved monitoring of the response of native species to alien plant control is also needed to justify efforts to control invasive alien plants.

As has occurred with habitat-fragmentation theory, emphasis needs to shift towards determining the types of native species that are most likely to go extinct (e.g. Fischer and Lindenmayer 2007), instead of debating the cause. Such a change in emphasis is likely to have significant positive consequences for the conservation of native species threatened by alien plant species. We hope that the six thresholds on the extinction trajectory along with the issues associated with determining extinction outlined in this paper will aid this transition by emphasizing the importance of other thresholds besides the end point—extinction.

## Sources of Funding

P.O.D. received funding from the DST-NRF Centre of Excellence for Invasion Biology (CIB) as a CIB Fellow, for which he is extremely grateful. P.O.D. also received funding and support from the University of Canberra to undertake the CIB Fellowship. D.M.R. acknowledges support from the CIB and National Research Foundation of South Africa (Grant 85417).

## Contributions by the Authors

P.O.D. conceived the basis of the framework. Independently, D.M.R. conceived the need for the paper and developed some aspects of the framework. P.O.D. and D.M.R. collaborated to refine the framework and structure the paper. P.O.D. led the writing and fine-tuning of the framework with major contributions from D.M.R. throughout the process.

## Conflicts of Interest Statement

No conflicts of interest.
